# Cost-effectiveness of daclatasvir plus asunaprevir for chronic hepatitis C genotype 1b treatment-naïve patients in China

**DOI:** 10.1371/journal.pone.0195117

**Published:** 2018-04-10

**Authors:** Yun Lu, Xiuze Jin, Cheng-a-xin Duan, Feng Chang

**Affiliations:** School of International Pharmaceutical Business, China Pharmaceutical University, Nanjing, Jiangsu, China; Centers for Disease Control and Prevention, UNITED STATES

## Abstract

**Background:**

Hepatitis C is the second fastest growing infectious disease in China. The standard-of-care for chronic hepatitis C in China is Pegylated interferon plus ribavirin (PR), which is associated with tolerability and efficacy issues. An interferon- and ribavirin-free, all-oral regimen comprising daclatasvir (DCV) and asunaprevir (ASV), which displays higher efficacy and tolerability, has recently been approved in China.

**Objectives:**

This study is to estimate the cost-effectiveness of DCV+ASV (24 weeks) for chronic hepatitis C genotype 1b treatment-naïve patients compared with PR regimen (48 weeks) in China.

**Methods:**

A cohort-based Markov model was developed from Chinese payer perspective to project the lifetime outcomes of treating 10,000 patients with an average age of 44.5 with two hypothetical regimens, DCV+ASV and PR. Chinese-specific health state costs and efficacy data were used. The annual discount rate was 5%. Base-case analysis and sensitivity analysis were conducted.

**Results:**

For HCV Genotype 1b treatment-naïve patients, DCV+ASV proved to be dominant over PR, with a cost saving of ¥33,480(5,096 USD) and gains in QALYs and life years of 1.29 and 0.85, respectively. The lifetime risk of compensated cirrhosis, decompensated cirrhosis, hepatocellular carcinoma and liver-related death was greatly reduced with DCV+ASV. Univariate sensitivity analysis demonstrated that key influencers were the discount rate, time horizon, initial disease severity and sustained virological response rate of DCV+ASV, with all scenarios resulting in additional benefit. Probabilistic sensitivity analysis demonstrated that DCV+ASV has a high likelihood (100%) of being cost-effective.

**Conclusion:**

DCV+ASV is not only an effective and well-tolerated regimen to treat chronic HCV genotype 1b infection treatment-naïve patients, but also is more cost-effective than PR regimen. DCV+ASV can benefit both the public health and reimbursement system in China.

## Introduction

Hepatitis C is a liver disease caused by hepatitis C virus which is mainly transmitted by blood. Studies show that 60% to 85% of patients with hepatitis C will develop chronic Hepatitis C (CHC) infection [1]. Patients with CHC are under the risk of fibrosis progression and advanced liver-related complications such as compensated cirrhosis (CC), decompensated cirrhosis (DC), hepatocellular carcinoma (HCC) and liver-related death, which leads to substantial health and economic burden for both patients and the society [2].

In China, recent epidemiological studies suggest that the reporting incidence of HCV infection has risen from 0.7 to 15.0 cases per 100,000 persons over the last decade [3]. The overall prevalence of chronic HCV infection is estimated to be 0.2%-1.2%, and the total number of persons chronically infected is approximately 8.9 million [4]. The estimated age-standardized rates of HCC incidence cases in China is 22.3 per 100,000 persons [5]. While chronic HBV remains the dominant cause of liver disease in China [6], HCV currently accounts for a relatively small but increasing proportion of HCC [7], with age-standardized death rates increasing 1.5 per 100,000 population from 1990 to 2010 [8]. The principle genotype of HCV in China is genotype 1b (56.8%), followed by genotype 2(24.1%) and genotype 3(9.1%) [9].

For a long time, pegylated interferon plus ribavirin (PR) for 48 weeks has been the standard-of-care for HCV in China [9], which has suboptimal efficacy and safety. Its sustained virological response (SVR) rates are relatively low: 71.1% overall and 62.4% among genotype 1b patients [10], and it has varieties of side effects, leading to numerous contraindications and poor tolerability [11]. A novel, interferon-free regimen comprising daclatasvir (DCV) and asunaprevir (ASV) has recently been approved in China, providing optimal choice for Chinese patients. This direct-acting antiviral (DAA) regimen, with improved SVR rate and tolerability over PR regimen [12], can achieve SVR rates higher than 90% and be administered orally with shorter treatment duration (24 weeks) [13]. According to the latest guideline, immediate treatment with DAA is recommended if patients could afford medical expenses during the course of treatment [9].

This study aims to estimate the cost-effectiveness of DCV+ASV (24 weeks) compared with PR regimen (48 weeks) for the treatment of HCV genotype 1b treatment-naïve Chinese patients.

## Materials and methods

### Model structure and assumptions

**Fig 1:**
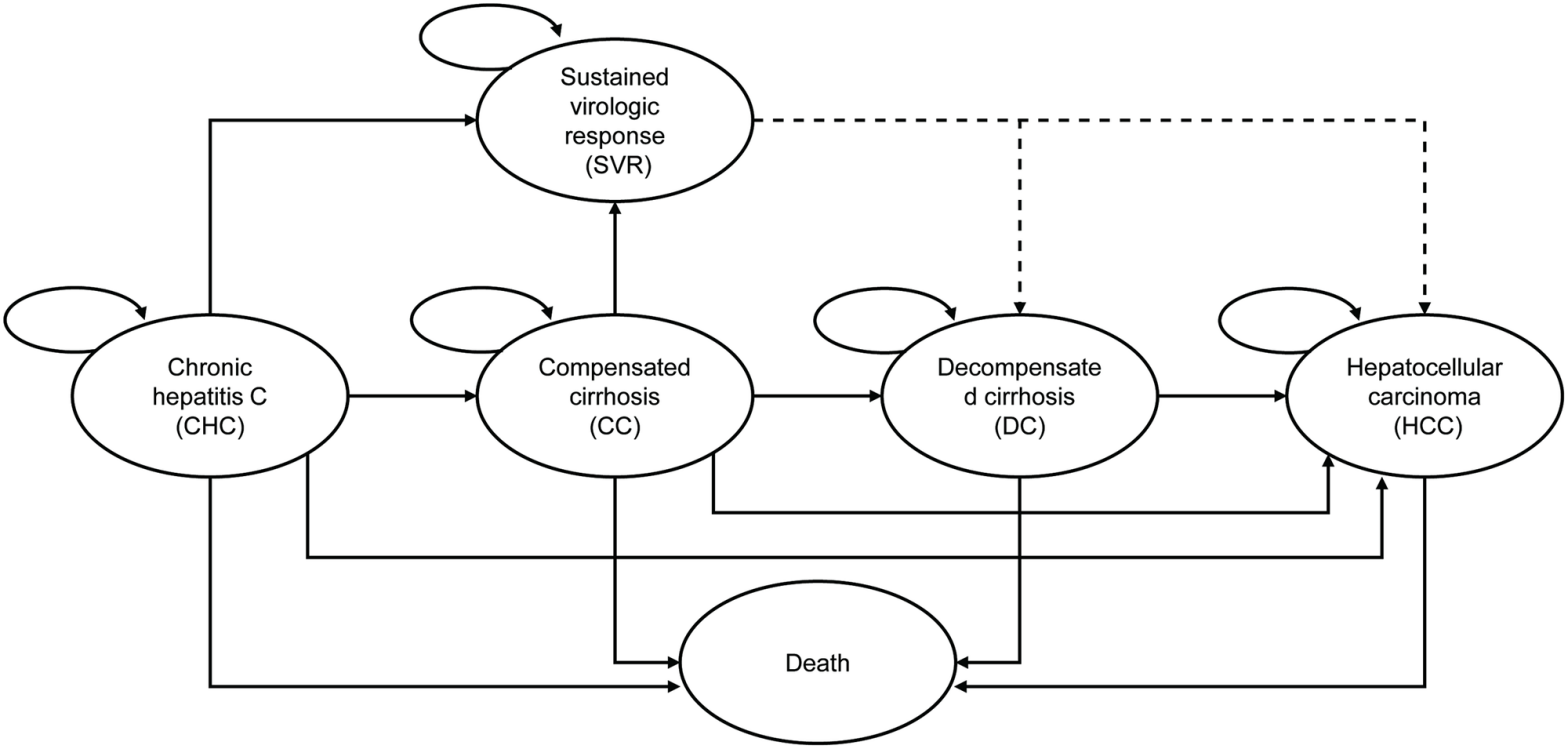
Structure of the Markov model.

In this study, an established Markov model [14–16](Fig 1), developed in Microsoft Excel 2013, was used from Chinese payer perspective to estimate the lifetime outcomes of treating a cohort of HCV genotype 1b treatment-naïve patients with two hypothetical regimens, DCV+ASV (24 weeks) and PR (48 weeks). The model runs in annual cycles over a lifetime horizon (up to 80 years) and an annual discount rate of 5% was applied.

10,000 HCV genotype 1b treatment-naïve patients were defined and they entered the model at either CHC or CC disease stage. Patients moved to the SVR health state if they had undetectable HCV RNA 12 or 24 weeks after the treatment. It was assumed that, if SVR was achieved, the default model setting was to halt disease progression. The model allowed the modeling of continued progression in patients that achieved SVR from the compensated cirrhosis stage. Patients who failed to achieve SVR were likely to keep their health state, or progress to DC, HCC or death.

### Baseline cohort characteristics

Baseline cohort characteristics included mean age, male proportion, and initial disease severity. These data were collected from a previous Chinese study [17]. A hypothetical cohort of 10,000 genotype 1b treatment-Naïve patients aged 44.5 years entered the model. Among the cohort members, 52.10% were male; 93.90% had chronic hepatitis C without cirrhosis and the remaining 6.10% had compensated cirrhosis (Table 1). Coinfection with HIV or HBV were excluded from the model.

**Table 1:** Baseline cohort characteristics.

Baseline characteristics	Mean	SE	Distribution	Source
Age (years)	44.5	0.53	Normal	Rao et al, 2014 [17]
Proportion male (%)	52.10%	2.07%	Beta	
CHC (%)	93.90%	1.03%	Beta	
CC (%)	6.10%	1.03%	Beta	

CHC: chronic hepatitis C; CC: compensated cirrhosis; SE: standard error

### Treatment strategies and clinical inputs

According to the latest guideline in China, DCV+ASV for 24 weeks without ribavirin was recommended for treating HCV genotype 1b naïve patients with or without compensated cirrhosis [9]. As a reference, PR for 48 weeks served as a comparator regimen. The goal of HCV treatment is to eradicate virus, and its accepted clinical endpoint is SVR, defined by undetectable HCV RNA for 12 (SVR12) or 24 weeks (SVR24) after the treatment [18]. Patients with a decrease of HCV RNA<2log10 IU/mL at week 12 discontinued treatment as a standard futility rule [11].

Treatment-related inputs specific to treatment-naïve patients with HCV genotype 1b were applied as follows:

PR: SVR = 62.4%; discontinuation rate: 3.9% [10]

DCV+ASV: SVR = 92.4%; discontinuation rate:0.6% [19]

### Modelling disease progression

Because well-designed, long-term prospective studies in patients with chronic hepatitis C or compensated cirrhosis in China are not available, the annual transition probabilities were derived from a 2013 literature review undertaken by the Japanese Ministry for Health, Labour and Welfare [20], as is presented in Table 2.

**Table 2:** Annual health state transition rates.

Health states transition		Rate	SE	Source
Chronic hepatitis C transition rates	CHC→CC	0.06	0.007	MHLW, 2013 [20]
	CHC→HCC	0.007	0.004	
	CC→DC	0.041	0.002	
	CC→HCC	0.019	0.002	
Complication transition rates	DC→HCC	0.024	0.004	
	DC→Death(1^st^ year)	0.142	0.011	
	DC→Death(2^nd^ year+)	0.142	0.011	
	HCC→Death(1^st^ year)	0.576	0.036	
	HCC→Death(2^nd^ year+)	0.576	0.036	
Post-SVR progression	Post SVR(CC)→DC	0	0	Assumption
	Post SVR(CC)→HCC	0	0	

CHC: chronic hepatitis C; CC: compensated cirrhosis; DC: decompensated cirrhosis; HCC: hepatocellular carcinoma; SE: standard error; SVR: sustained virological response

Mortality of decompensated cirrhosis and HCC were modeled via static independent transition rates reported in Table 2. Mortality of chronic hepatitis C, compensated cirrhosis and SVR were modelled according to annual all-cause mortality sourced from published China life tables [21].

### Health state utility values

Health-related quality of life (HRQoL) was used to indicate preferences for particular health outcomes [22]. It has been demonstrated that quality of life is reduced when disease progression occurs, and patients with more advanced fibrosis or cirrhosis suffer greater impairment [23–24]. Although symptoms can be mild during the early stages of chronic infection, there can be a reduction in quality of life, due to some non-specific symptoms such as tiredness, malaise and cognitive impairment [24]. It has also been observed that the awareness of carrying a transmissible disease and the perceived risk of passing the disease to others can significantly affect the quality of life in patients [24]. In the absence of China-specific inputs, health state utility values were obtained from a 2013 literature review undertaken by the Japanese Ministry for Health, Labour and Welfare (MHLW). Future utility values were discounted at 5% per year. All the utilities are presented in Table 3.

**Table 3:** Annual health state utility values.

Health state	Mean	SE	Source
Chronic hepatitis C	0.86	0.02	MHLW, 2013 [20]
Compensated cirrhosis	0.77	0.02	
Decompensated cirrhosis	0.64	0.04	
HCC	0.47	0.05	
SVR from chronic hepatitis C and compensated cirrhosis stages	0.93	0.03	

HCC: hepatocellular carcinoma; SE: standard error; SVR: sustained virological response

### Disease and treatment costs

This study is from the payer perspective, thus the costs only include direct medical costs, which come from treatment acquisition cost and health state cost. The treatment acquisition cost was collected via fixed official public resources which does not need validation. For DCV+ASV, weekly drug cost is ¥2,408.75(366.61 USD) (daclatasvir and asunaprevir are ¥2,185.75(332.67 USD) and ¥223(33.94 USD), respectively). A one-off mutation testing (¥334 or 50.83 USD) is applied at the start of treatment with DCV+ASV. For PR regimen, the weekly cost of pegylated interferon, ribavirin is ¥1,124(171.07 USD) and ¥21(3.20 USD) respectively. Treatment-related adverse events were not considered in this analysis due to a lack of data and its likely non-significant effect upon cost-effectiveness outcomes. Moreover, the cost of monitoring is included in health state costs. The average costs for each health state are collected from published literature. A survey found that the more advanced the disease progress, the higher costs to the condition are [25]. Since liver transplant is not possible or appropriate for most patients [26], its cost is not included in this study. Patients who achieved SVR were assumed not to incur any further HCV-related direct medical costs. Future costs were discounted at 5% per year. The health state costs are presented in Table 4.

**Table 4:** Annual health state costs.

Health state	Mean cost, RMB (USD)	SE, RMB (USD)	Distribution	Source
Chronic hepatitis C	5714.61† (869.76†)	944.32 (143.73)	Gamma	Chen et al, 2016 [27]
Compensated cirrhosis	16265.14 (2475.55)	5357.49 (815.41)	Gamma	
Decompensated cirrhosis	36225.78 (5513.56)	7252.48 (1103.83)	Gamma	
HCC	76464.88 (11637.95)	10958.21 (1667.84)	Gamma	
SVR from compensated cirrhosis§	11531.99 (1755.17)	3798.46 (578.13)	Gamma	

HCC: hepatocellular carcinoma; SE: standard error; SVR: sustained virologic response

1 RMB = 0.1522 USD

^†^ Applied in the first year only, based upon clinical expert opinion

^§^ Assumed that no direct medical costs incurred by patients following SVR in chronic hepatitis C

### Model outcomes

Clinical outcomes of the model include the number of complications (CC, DC, HCC and liver-related death), life years and quality-adjusted life years (QALYs) gained. Economic outcomes include total costs per patient. Incremental cost-effectiveness ratio (ICER) of DCV+ASV compared with PR regimen was calculated.

### Sensitivity analysis

Univariate sensitivity analysis was performed to assess the influence of selected variables in the base case and their impact on the model results. The key parameters varied as follows: simulation time horizon (10 and 20 years), discounting rate (0% and 6%), the starting state of patients (CHC only and CC only), mean age (±10 years), male/female proportion (all male and all female), disease state costs (±20%), disease state health utility (±20%), disease progression rates (±20%), inclusion of disease progression from compensated cirrhosis state following SVR (CC_SVR_→DC = 0.001; CC_SVR_→HCC = 0.008) [28], SVR of two regimens (±20%), exclusion of discontinuation.

Probabilistic sensitivity analysis (PSA) was undertaken, in which model input values were sampled from distributions around the mean of base case inputs. PSA is conducted to estimate the overall uncertainty in results predicted by utilizing input parameters. Standard errors and distribution types for the sampled input parameters have been reported in relevant tables above.

## Results

### Base-case analysis

The base-case analysis suggested that DCV+ASV was estimated to be dominant over PR, with a cost saving of ¥33,480(5,096 USD) and gains in QALYs and life years of 1.29 and 0.85, respectively (Table 5).

**Table 5:** Base-case analysis: Cost-effectiveness of DCV+ASV versus PR.

Regimen	Cost, RMB (USD)	Life Years	QALYs	Cost/Life Year, RMB (USD)	Cost/QALY, RMB (USD)
DCV+ASV	748,528,847 (113,926,091)	158,915	146,253	-	-
Per Patient	74,853 (11,393)	15.89	14.63		
PR	1,083,326,336 (164,882,268)	150,400	133,378	-	-
Per Patient	108,333 (16,488)	15.04	13.34		
Total Difference	-334,797,489 (-50,956,178)	8,515	12,875	Dominant	Dominant
Per Patient	-33,480 (-5,096)	0.85	1.29		

1 RMB = 0.1522 USD

**Fig 2:**
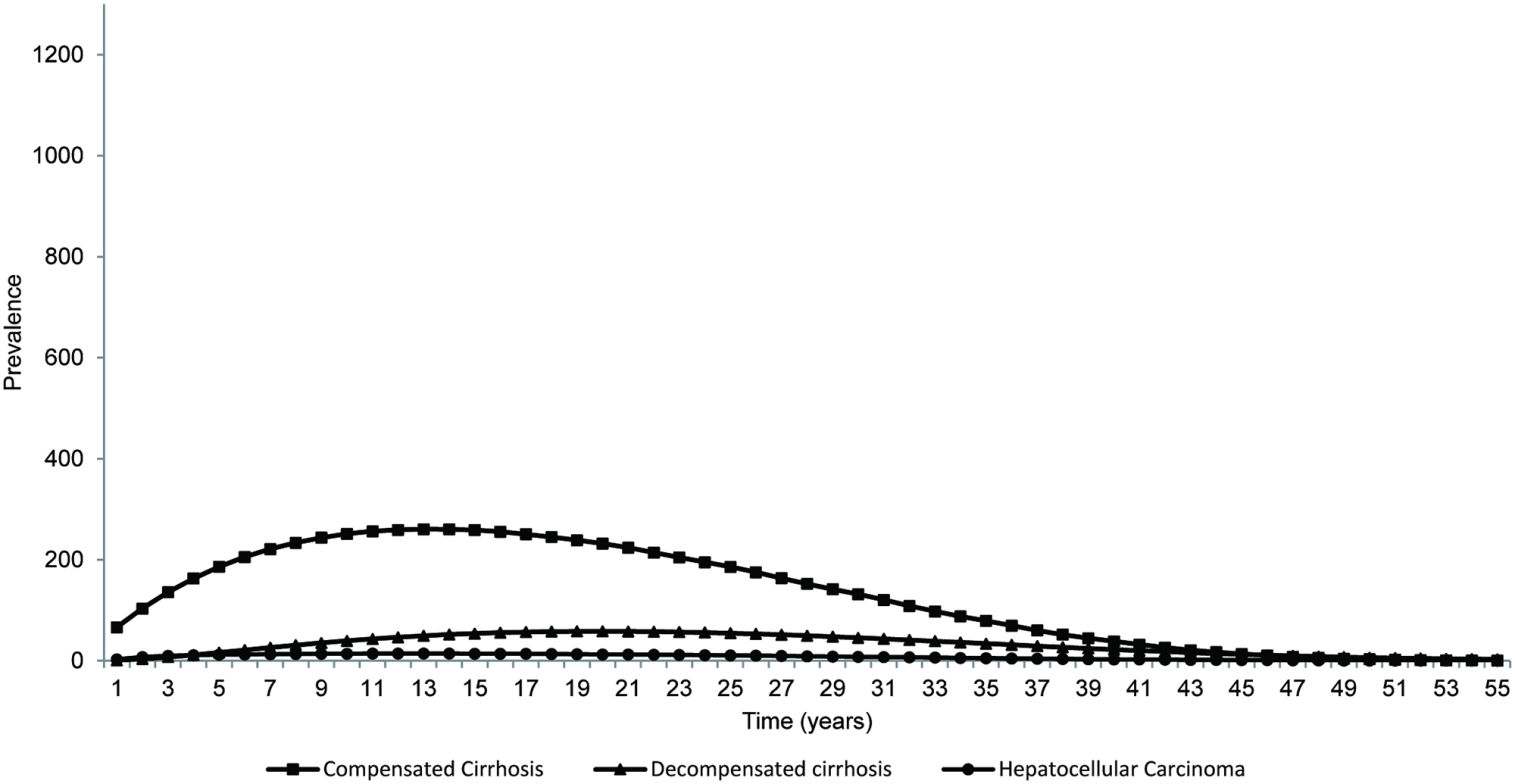
Base-case analysis: Estimated incidence of HCV-related complications (compensated cirrhosis, decompensated cirrhosis, and hepatocellular carcinoma) after treating with DCV+ASV.

**Fig 3:**
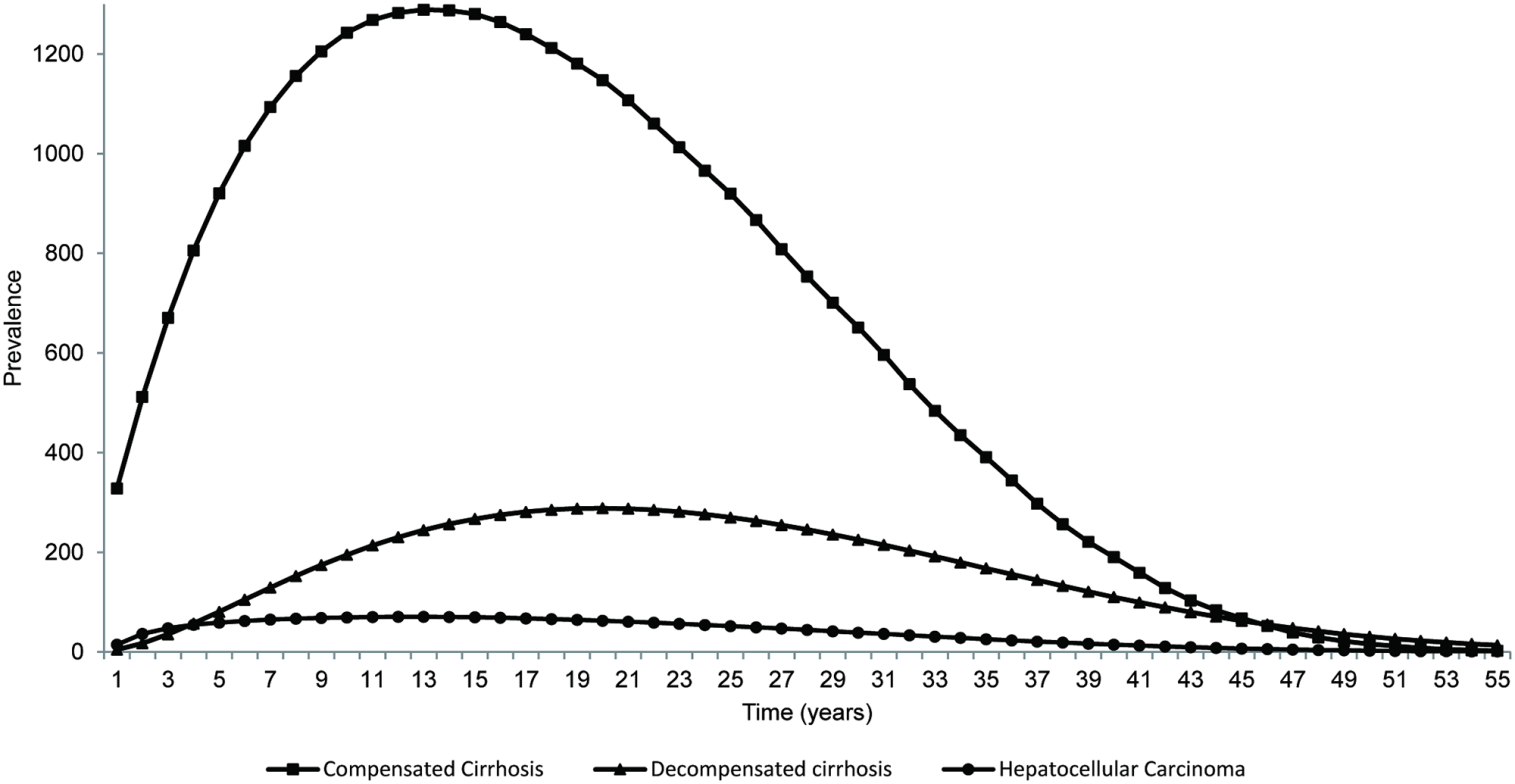
Base-case analysis: Estimated incidence of HCV-related complications (compensated cirrhosis, decompensated cirrhosis, and hepatocellular carcinoma) after treating with PR.

Because of higher efficacy seen with DCV+ASV, the lifetime risks of CC, DC, HCC and liver-related death were greatly reduced in this treatment arm (Table 6). Figs 2 and 3 present a graphical interpretation of the prevalence of HCV-related complications of patients treated with different regimens, and further demonstrate the significance of the observed event reductions associated with DCV+ASV.

**Table 6:** Base-case analysis: Lifetime risk of complications after treating with DCV+ASV and PR.

Regimen	CC	DC	HCC	Liver-related Death
DCV+ASV	528.79	288.83	239.43	483.90
PR	2616.11	1428.94	1184.56	2394.05
Difference	-2087.33	-1140.11	-945.12	-1910.15

### Sensitivity analysis

**Fig 4:**
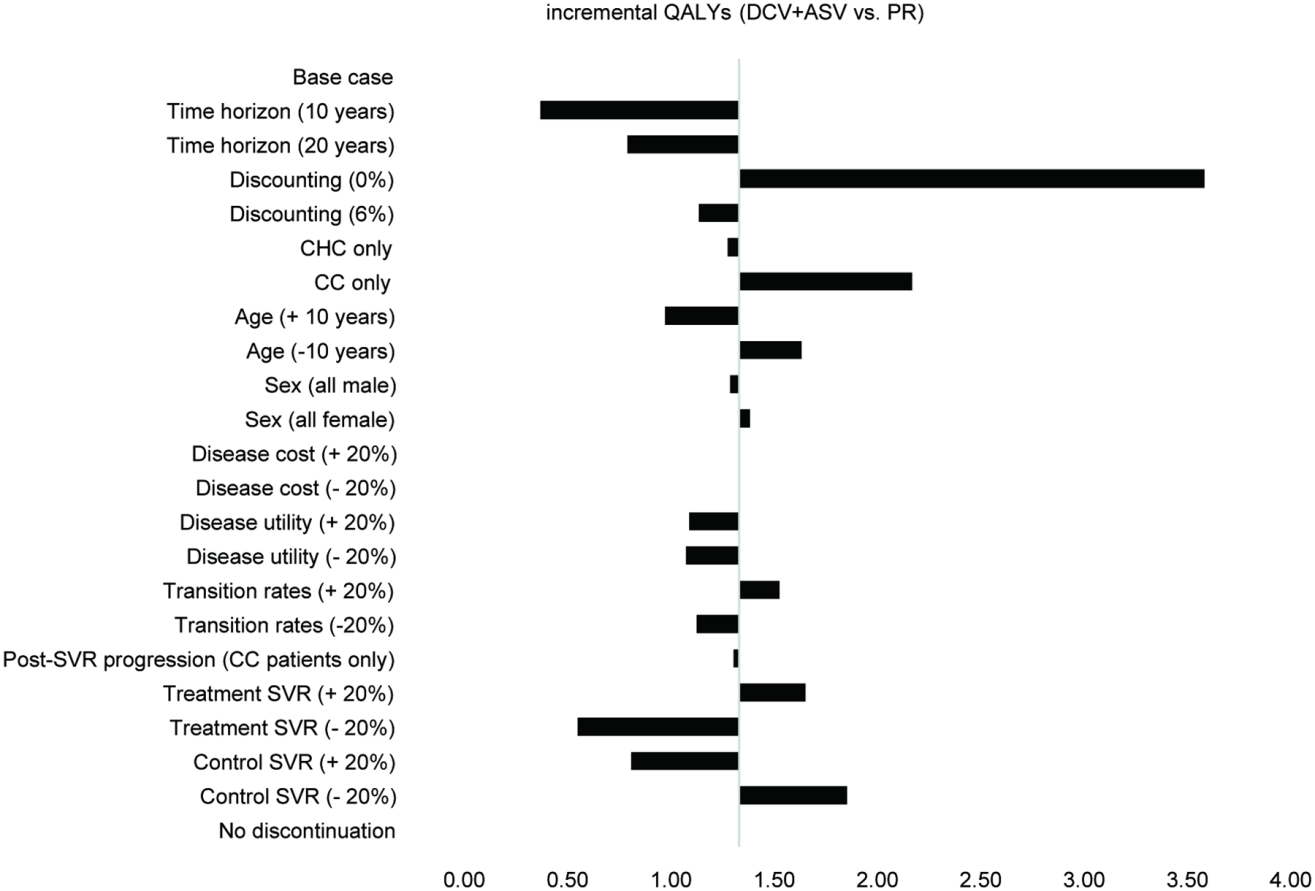
Univariate sensitivity analysis.

Univariate sensitivity analysis (Fig 4) demonstrated that key influencers were the discount rate, time horizon, patients’ initial disease severity and SVR rate of DCV+ASV, with all scenarios resulting in additional benefit.

**Fig 5:**
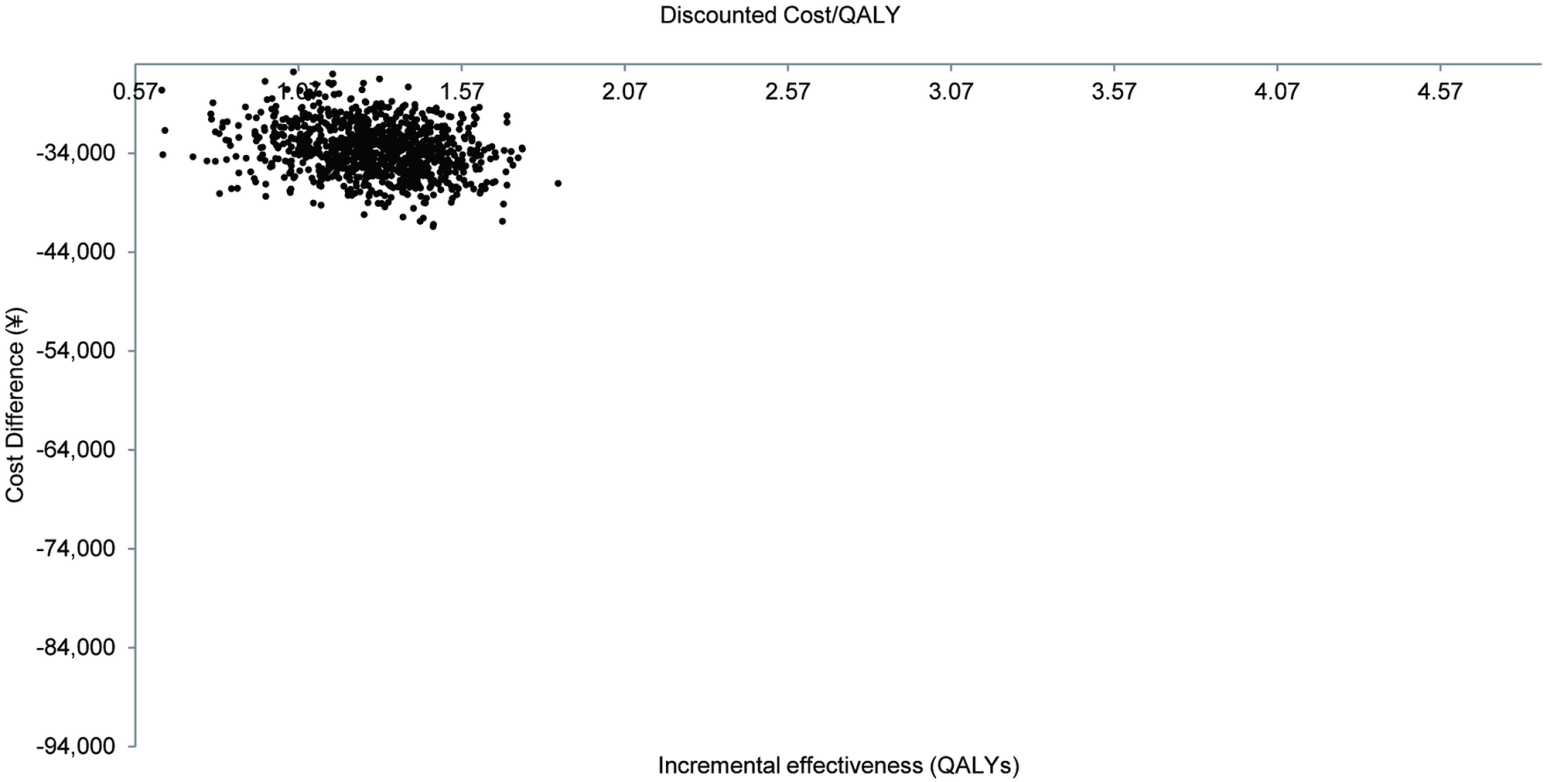
Probabilistic sensitivity analysis: Cost-effectiveness scatter plots.

In the PSA, 10,000 Monte Carlo simulations were conducted. Assuming payers have a willingness-to-pay threshold of RMB 50,251(7,648 USD) (GDP per capita of 2015) per QALY, DCV+ASV has a high likelihood (100%) of being cost-effective (Fig 5).

## Discussion

The novel interferon- and ribavirin-free direct-acting antiviral (DAA) regimen, DCV+ASV for the treatment of chronic HCV genotype-1b, with a shorter treatment duration than the current standard-of-care (24 weeks vs. 48 weeks) in China, has just been approved by China Food and Drug Administration. Due to the large population of patients infected with HCV in China, the demand for the new therapy is exponentially increasing over time. Immediate application of DAA is recommended if patients could afford medical expenses during the course of treatment [9]. To well identify the cost-effectiveness of DCV+ASV over PR is of great importance in decision-making processes such as determining its affordability and the possibility of reimbursement.

In this study, the ICER representing the comparison of the two regimens was calculated by using a validated cohort-based Markov lifetime model designed to simulate the natural history of chronic hepatitis C and its complications. The model results demonstrated that DCV+ASV was clearly dominant over PR, with a cost saving of ¥33,480(5,096 USD) and gains in QALYs and life years of 1.29 and 0.85 respectively, and end-stage liver disease complications and liver-related mortalities were significantly reduced at the same time. Additionally, shorter duration of therapy (24 weeks vs 48 weeks) is also a positive factor for choosing DCV+ASV. The univariate sensitivity analysis and probabilistic sensitivity analysis both confirmed the model results.

Our study has several limitations. Firstly, we did not consider the role of Traditional Chinese Medicine (TCM) in hepatitis C treatment in this study. Actually, TCMs, such as glycyrrhizic acid, silymarin, and bicyclol, are widely added to PR regimen as adjuvant therapy to improve symptoms in China. Because of the potential drug-drug interactions, TCM is no longer recommended to be used with DAAs. It means that the cost of PR regimen is actually higher than the result of the model simulation, which further confirms the cost-effectiveness of DCV+ASV. However, the application of TCMs in the treatment of hepatitis C has not been studied in clinical trials, so there is no powerful data to prove that TCMs can effectively eliminate the hepatitis C virus. In order to reduce the uncertainty of model results, the cost of TCM was excluded from the Markov model. Secondly, due to our lack of local clinical data of the progression from chronic hepatitis C to end-stage liver disease and mortality, the annual health state transition probabilities were obtained from a Japanese study. Thirdly, since no China-specific data for HCV-related mortality are available, all-cause mortality probabilities were used to model mortality from chronic hepatitis C, compensated cirrhosis and SVR health states without adjustment (i.e. to remove HCV-related mortality from all-cause mortality in the general population). It is assumed that life expectancy for individuals achieving SVR is equivalent to the general population. This approach has the potential to double count HCV deaths in those who remain infected. However, as the proportion of deaths caused by HCV among the general population is likely to be very small, no significant bias would be introduced by this approach, nor would it have a significant impact on results. Fourthly, in the base case analyses, we assumed that once a patient has achieved SVR, then it is no longer possible for them to incur disease progression. Nevertheless, a one-way sensitivity analysis was conducted to test the impact of the model containing post-SVR progression from the compensated cirrhosis disease state. What’s more, the treatment-related adverse events were not considered in this study due to the lack of data.

## Conclusions

DCV+ASV is a regimen not only effective and well-tolerated to treat chronic HCV genotype 1b infection treatment-naïve patients, but also more cost-effective than PR regimen for the public health and reimbursement system in China.

## Supporting information

S1 File Markov progress of treatment arm (DCV+ASV) and control arm (PR). (XLSX)

S1 Table Non-discounted cost-effectiveness of DCV+ASV versus PR. (DOCX)
