# Supplementary material for: Cost-effectiveness of daclatasvir plus asunaprevir for chronic hepatitis C genotype 1b treatment-naïve patients in China
Source: PLoS One. 2018 Apr 10;13(4):e0195117. doi: 10.1371/journal.pone.0195117 (PMC5892899; doi:10.1371/journal.pone.0195117)
Supplement: S1 Table — (DOCX) [file pone.0195117.s002.docx]

**S1 Table. Non-discounted cost-effectiveness of DCV+ASV versus PR**

| Regimen | Cost, RMB (USD) | Life Years | QALYs | Cost/Life Year, RMB (USD) | Cost/QALY, RMB (USD) |
| --- | --- | --- | --- | --- | --- |
| DCV+ASV | 972,386,398 (147,997,210) | 321,650 | 296,394 | - | - |
| Per Patient | 97,239 (14,800) | 32.16 | 29.64 |  |  |
| PR | 1,725,507,766 (262,622,282) | 294,020 | 260,996 | - | - |
| Per Patient | 172,551 (26,262) | 29.40 | 26.10 |  |  |
| Total Difference | -753,121,368 (-114,625,072) | 27,630 | 35,398 | Dominant | Dominant |
| Per Patient | -75,312 (-11,462) | 2.76 | 3.54 |  |  |

1 RMB=0.1522 USD
